# Clinical Findings of Melanoma-Associated Retinopathy with anti-TRPM1 Antibody

**DOI:** 10.1155/2021/6607441

**Published:** 2021-09-08

**Authors:** Yoichiro Shinohara, Ryo Mukai, Shinji Ueno, Hideo Akiyama

**Affiliations:** ^1^Department of Ophthalmology, Gunma University School of Medicine, Maebashi, Gunma, Japan; ^2^Department of Ophthalmology, Nagoya University School of Medicine, Nagoya, Aichi, Japan

## Abstract

**Introduction:**

We report the clinical features and clinical course of melanoma-associated retinopathy (MAR), in which autoantibodies against the transient receptor potential cation channel subfamily M member 1 (TRPM1) were detected. *Case Presentation*. A 74-year-old man was referred to our hospital for treatment of bilateral vision loss. The best-corrected visual acuity was 20/100 in the right eye and 20/200 in the left eye. His electroretinogram (ERG) showed a reduced b-wave and a normal dark-adapted a-wave in both eyes. Optical coherence tomography (OCT) revealed loss of the interdigitation zone in both eyes. We strongly suspected MAR based on the markedly reduced b-wave in the ERG and a history of intranasal melanoma. The diagnosis was confirmed after autoantibodies against TRPM1 were detected in his blood serum. Fifteen months later, his ERG remained unchanged, and OCT showed bilateral cystic changes in the internal nuclear layer. The visual acuity in both eyes also remained unchanged.

**Conclusions:**

Anti-TRPM1 autoantibodies were detected in a patient diagnosed with MAR who had negative flash ERG and retinal microstructural abnormalities, and the impairment did not recover during the follow-up period. Identification of anti-TRPM1 antibodies was helpful in confirming the diagnosis of MAR.

## 1. Introduction

Melanoma-associated retinopathy (MAR) is a disease associated with melanoma that causes dysfunction of retinal ON-bipolar cells [1]. It is a rare disease in Japan, with a lower incidence than in Europe and the United States [2, 3]. Most patients with MAR have night blindness, photopsia, and nephelopsia, but the vision is usually preserved [4]. A negative-type electroretinogram (ERG) in which a normal a-wave and a reduction in the b-wave amplitude are detected is an essential examination for the diagnosis of MAR [5], although misalignment of the outer retinal microstructure in optical coherence tomography (OCT) is a supportive manifestation for the diagnosis. Transient receptor potential cation channel subfamily M member 1 (TRPM1) is an mGluR6-coupled ion channel in the retinal ON-bipolar cell signal transduction pathway [6] and one of the target antigens for patients with MAR [7]. Herein, we report a case of anti-TRPM1 antibody-positive MAR with visual impairment that followed the clinical course, including OCT findings.

## 2. Case Presentation

**Figure 1:**
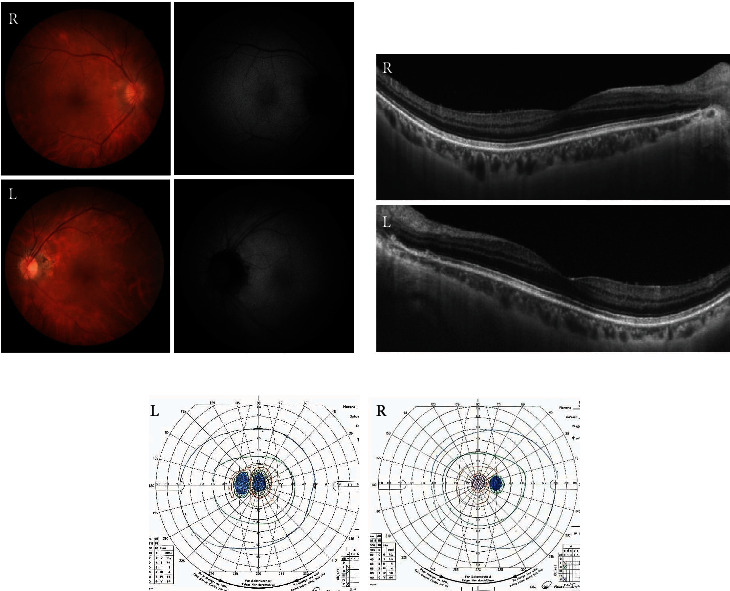
Ophthalmological findings of a 74-year-old male patient at the first visit. The visual acuities in the right and left eyes were 20/100 and 20/200, respectively. (a) Fundus photographs and fundus autofluorescence of the patient showing an almost normal fundus. (b) Swept-source optical coherence tomography image showing obscuration of the interdigitation lines in both eyes. (c) The Goldmann visual field test, showing central scotoma in both eyes.

**Figure 2:**
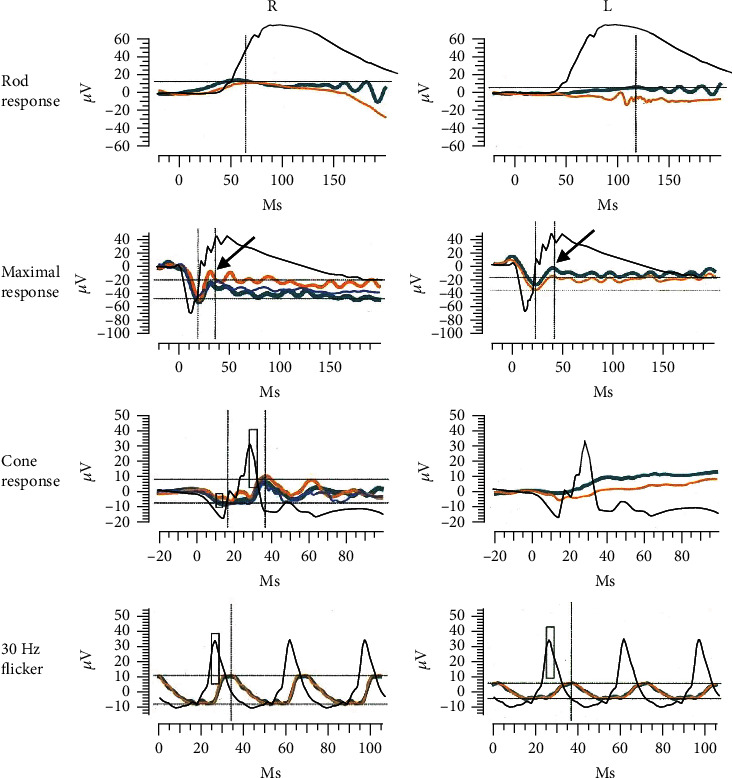
Full-field electroretinogram (ERG) of the patient including the rod response, rod and cone mixed maximal response, and 30-Hz flicker response. Yellow, green and blue indicate the first, second, and third responses, respectively. Arrows in the maximal responses show negative-type ERGs in both eyes. Black indicates ERGs from a control eye without any retinal damage.

**Figure 3:**
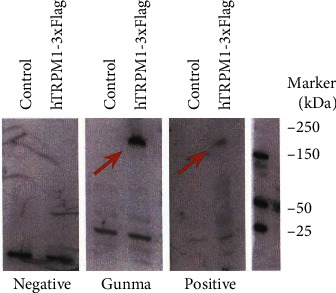
Western blot analysis of human TRPM1 antibody using serum from the patient with MAR. Red arrows indicate the hTRPM1-3xFlag protein bands.

A 74-year-old Japanese man gradually developed visual disturbances in both eyes. He was referred to our department four months after his symptoms emerged due to an unknown cause. At his first hospital visit, the best-corrected visual acuities of the right and left eyes were 20/100 and 20/200, respectively. Intraocular pressure was normal. Slit-lamp examination and fundus photography yielded normal results in both eyes (Figure 1(a)). Swept-source optical coherence tomography (SS-OCT; DRI OCT-1 Triton, Topcon Corp., Tokyo, Japan) showed loss of interdigitation lines in both eyes (Figure 1(b)). The Goldmann visual field test showed central scotoma in both eyes (Figure 1(c)). MRI showed no specific abnormalities. The patient had been diagnosed with prostate cancer 9 years ago, and enzalutamide was administered for bone metastases at the initial visit. He had been diagnosed with intranasal melanoma 2 years ago and underwent tumor resection and cervical lymph node dissection. We suspected cancer-associated retinopathy (CAR) based on his medical history, but the anti-recoverin antibody was negative. ERGs were recorded using the RETeval system (LKC Technologies, Gaithersburg, MD, USA) according to the standards of the International Society for Clinical Electrophysiology of Vision. The implicit times and amplitudes of a- and b-waves were automatically analyzed using the software integrated into the RETeval system. The full-field ERGs recorded during the first visit are shown in Figure 2. The rod responses were extinguished, and the rod and cone mixed maximal responses were negative-type ERGs having an a-wave with a normal amplitude and a b-wave with a weaker amplitude than the a-wave in both eyes. The cone responses in the right eye had a square-shaped a-wave and a reduced b-wave amplitude and prolonged implicit time, and those in the left eye were extinguished. The ERGs indicated that the function of retinal ON-bipolar cells was impaired [8]. The amplitudes of the 30-Hz flicker ERGs were almost normal but delayed. Since we suspected MAR from the patient's medical history and ERGs, we used western blot analysis to examine whether anti-TRPM1 antibodies were present in his serum. Serum examinations for the anti-TRPM1 antibody were performed at Nagoya University, as reported previously [9]. Because the autoantibodies against TRPM1 were positive in this patient's blood sample (Figure 3), he was diagnosed with MAR.

**Figure 4:**
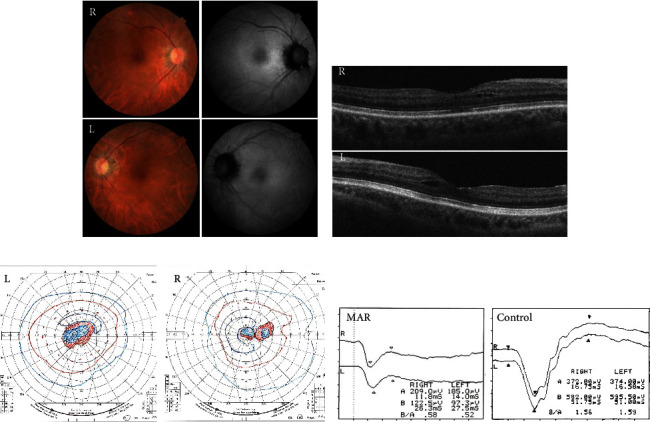
Ophthalmological findings of the patient 15 months after the initial visit. The visual acuities in the right and left eyes were 20/120 and 20/200, respectively. (a) Fundus photographs and fundus autofluorescence of the patient showing an almost normal fundus. (b) Optical coherence tomography image showing cyst-like lesions in the internal nuclear layer in both eyes. (c) The Goldmann visual field test, showing a slightly enlarged central scotoma in both eyes. (d) The negative-type waveform with reduced amplitude of the b-wave is unchanged in the maximal response of full-field electroretinogram.

Fifteen months later, the best-corrected visual acuity of both eyes remained unchanged. OCT demonstrated obscuration of the interdigitation lines and cystic changes in the internal nuclear layer (INL) in both eyes (Figures 4(a), 4(b)). The cystic lesions were not detected until the last visit. ERG and Goldmann visual field test results in both eyes essentially remained unchanged for 15 months (Figures 4(c), 4(d)).

## 3. Discussion

We present a case of MAR with negative flash ERG and retinal microstructural abnormalities. Identification of the anti-TRPM1 antibody was helpful in confirming the diagnosis of MAR. The patient was followed up for 15 months, and his visual acuity in one eye was preserved, although it deteriorated slightly in the contralateral eye.

According to previous reports in which changes in visual function were analyzed, visual acuity, color perception, and central visual field were generally preserved in most patients with MAR. However, in this case, the visual acuity of the patient was severely impaired at the initial visit and did not improve during the next 15 months. At the initial visit, SS-OCT was able to detect an irregularity in the interdigitation line, indicating photoreceptor damage, which did not recover during the follow-up period. This damage seemed to correlate with visual disturbances. TRPM1 was originally identified as a specific protein against melanocytes and has also been reported to be expressed in ON-bipolar cells as a binding protein against mGluR6 [6]. TRPM3 was expressed in high concentrations in the retinal pigment epithelium and inner retina. TRPM1 and TRPM3 have similar sequences [10], and the serum of patients with MAR may cross-react with TRPM1 and TRPM3 [11]. Thus, the patient's serum had a widespread effect on the retina, potentially leading to photoreceptor damage. This response may explain the visual disturbances detected in patients with MAR. At 15 months after the initial visit, OCT revealed a cystic change in the macula (Figure 4(b)). This lesion lies in the INL where bipolar cells are distributed, and it is possible that direct binding of anti-TRPM1 antibody to bipolar cells impairs these cells and leads to cystic changes in the INL. Although fundus fluorescein angiography was not performed in this patient, some CAR patients have been reported to have macular edema due to retinal vasculitis [12]. There was a difference in the ERG amplitude between both eyes of the patient (Figures 2 and 4(d)). All ERGs were performed under similar conditions, and the ERG amplitude may be correlated with the degree of retinal damage. Retinal damage that could not be assessed by analyzing the retinal structures may be more severe in the left eye than in the right eye of the patient.

Previously, our team reported a correlation between the detection of anti-TRPM1 antibodies and the clinical characteristics of paraneoplastic retinopathy with ON-bipolar cell dysfunction [7]. In this report, autoantibodies against TRPM1 were detected in 5 of the 10 cases. Among these, two had melanoma, and three had lung cancers. Interestingly, in one of the three cases with lung cancer, the negative b-wave and ERG at the initial visit dramatically recovered during the 1-year follow-up. However, in the other two cases, a deteriorated or unchanged b-wave was recorded at the end of the follow-up.

The differences in the clinical courses of each patient can be explained by the variability of recognition sites in anti-TRPM1 antibodies. Some recognition sites can bind to only one specific site, and the binding possibly causes only transient dysfunction of retinal ON-bipolar cells, while other recognition sites can cause severe damage to retinal ON-bipolar cells [7, 13].

In conclusion, this study shows the clinical course of a patient diagnosed with MAR who tested positive for anti-TRPM1 antibodies. In this case, both visual disturbance and retinal microstructural abnormalities at the initial visit did not recover for 15 months. Anti-TRPM1 antibodies may be useful for confirming the diagnosis of MAR; however, it is important to know the variety of clinical courses in each case with anti-TRPM1 antibodies.

## References

[1] Berson E. L., Lessell S. (1988). Paraneoplastic night blindness with malignant melanoma. *American Journal of Ophthalmology*.

[2] Chang A. E., Karnell L. H., Menck H. R. (1998). The National Cancer Data Base report on cutaneous and noncutaneous melanoma: a summary of 84,836 cases from the past decade. The American College of Surgeons Commission on Cancer and the American Cancer Society. *Cancer*.

[3] Murayama K., Takita H., Kiyohara Y., Shimizu Y., Tsuchida T., Yoneya S. (2006). Melanoma-associated retinopathy with unknown primary site in a Japanese woman. *Nippon Ganka Gakkai Zasshi*.

[4] Keltner J. L., Thirkill C. E., Yip P. T. (2001). Clinical and immunologic characteristics of melanoma-associated retinopathy syndrome: eleven new cases and a review of 51 previously published cases. *Journal of Neuro-Ophthalmology*.

[5] Dobson R., Lawden M. (2011). Melanoma associated retinopathy and how to understand the electroretinogram. *Practical Neurology*.

[6] Koike C., Obara T., Uriu Y. (2010). TRPM1 is a component of the retinal ON bipolar cell transduction channel in the mGluR6 cascade. *Proceedings of the National Academy of Sciences of the United States of America*.

[7] Ueno S., Inooka D., Nakanishi A. (2019). Clinical course of paraneoplastic retinopathy with anti-TRPM1 autoantibody in JAPANESE cohort. *Retina*.

[8] Kondo M., Sieving P. A. (2001). Primate photopic sine-wave flicker ERG: vector modeling analysis of component origins using glutamate analogs. *Investigative Ophthalmology & Visual Science*.

[9] Kondo M., Sanuki R., Ueno S. (2011). Identification of autoantibodies against TRPM1 in patients with paraneoplastic retinopathy associated with ON bipolar cell dysfunction. *PLoS One*.

[10] Brown R. L., Xiong W. H., Peters J. H. (2015). TRPM3 expression in mouse retina. *PLoS One*.

[11] Duvoisin R. M., Haley T. L., Ren G., Strycharska-Orczyk I., Bonaparte J. P., Morgans C. W. (2017). Autoantibodies in melanoma-associated retinopathy recognize an epitope conserved between TRPM1 and TRPM3. *Investigative Ophthalmology & Visual Science*.

[12] Rahimy E., Sarraf D. (2013). Paraneoplastic and non-paraneoplastic retinopathy and optic neuropathy: evaluation and management. *Survey of Ophthalmology*.

[13] Xiong W. H., Duvoisin R. M., Adamus G., Jeffrey B. G., Gellman C., Morgans C. W. (2013). Serum TRPM1 autoantibodies from melanoma associated retinopathy patients enter retinal on-bipolar cells and attenuate the electroretinogram in mice. *PLoS One*.

